# Integrated Sensing and Communication Using Random Padded OTFS with Reduced Interferences

**DOI:** 10.3390/s25154816

**Published:** 2025-08-05

**Authors:** Pavel Karpovich, Tomasz P. Zielinski

**Affiliations:** 1 Institute of Telecommunications, AGH University of Krakow, Al. Mickiewicza 30, 30-059 Krakow, Poland; karpovic@agh.edu.pl; 2 Nokia Solutions and Networks, 30-348 Kraków, Poland

**Keywords:** integrated sensing and communication (ISAC), OTFS modulation, random-padded OTFS, pilot-to-data interference, vehicle detection

## Abstract

The orthogonal time frequency space (OTFS) is a modulation designed to transmit data in high Doppler channels where the usage of the orthogonal frequency division multiplexing (OFDM) is challenging. The random padded OTFS (RP-OTFS) modulation, introduced recently, is an OTFS-like waveform optimized for more precise estimation of channel state information (CSI) and, in the case of integrated sensing and communication (ISAC), for radar detection as well. One of the main drawbacks of the RP-OTFS is the high level of interference between carriers (the inter-carrier interference—ICI) of Doppler-delay (DD) grid. In the article, we optimize the RP-OTFS waveform in terms of reducing the level of pilot-to-data interference and also offer a way to reduce the data carrier interference. The reduction in the pilot-to-data interference is achieved due to the introduction of the following: (1) redistributing interferences along the DD grid, and (2) special DD grid configuration. In turn, the reduction in data carrier interference is achieved by extrapolating the estimate of channel state information. The proposed approach allows us to reduce the influence of the interference component and, as a result, to improve the probability of correct demodulation in the ISAC RP-OTFS system. Various DD grid configurations for different use cases from a radar point of view are considered in the article. The questions of choosing appropriate values of the DD grid parameters depending on the operating environment are also discussed here. In simulations, the ICI-reduced RP-OTFS is compared with its predecessor, the regular RP-OTFS, and classical modulations: OFDM and zero-padded OTFS, and benefits of its usage are shown: lower bit error rate (BER) of the transmission and higher detection probability of the radar detection.

## 1. Introduction

As 5G communication systems were delivered in 2019, today the scientific community is focused on next-generation systems, called 6G. Currently (July 2025), there is no official specification for 6G from the 3GPP group, but there are a lot of publications on the vision of 6G systems—the most noticeable are [1,2]. There are different opinions on some aspects of 6G. At the same time, there are some common points in most of the articles: increasing the scale of massive multiple input multiple output (mMIMO) technology, the usage of intelligent reflecting surfaces (IRS) [3], a more reliable network with more spectral efficiency, the use of artificial intelligence (AI) technologies [4], etc., but the most important of the common points for new waveform research are higher operating frequencies and the emergence of integrated sensing and communication (ISAC) [5,6,7,8,9,10].

The increase in operating carrier frequency is the answer to the demand for an increasing data rate for potential 6G applications, for example, holographic video calls [2]. But this also brings new challenges. One of the main problems in physical layer processing is the Doppler effect. From a Doppler frequency shift point of view, the increase in the carrier frequency will further aggravate the situation, since the Doppler frequency shift is directly proportional to the carrier frequency. Orthogonal Frequency Division Multiplexing (OFDM) (de facto the standard waveform for most modern communication systems since the mid-1990s) assumes that the channel impulse response during the OFDM symbol does not change. This makes the usage of the OFDM waveform in a strong Doppler environment very problematic. For example, in 5G, special numerologies for frequency range 2 (FR2—above 24GHz) [11] were introduced to make OFDM symbols shorter and to adapt OFDM for a strong Doppler environment [12]. But this also has its own drawback: more bandwidth is occupied for the same number of subcarriers.

Moreover, the channel coherence time limits the length of the OFDM symbol, while on the other hand, the coherence bandwidth sets the spacing between subcarriers. In addition to this, considering that the bandwidth occupied by the signal is inversely proportional to its duration makes the situation even more contradictory.

To better understand this, let us consider the following example. Let the number of OFDM subcarriers be constant. To operate in channels with strong Doppler effect (short coherence time), we want to shorten the duration of the OFDM symbol while keeping the number of subcarriers constant. This means increasing the spacing between them. However, on the other hand, increasing the sub-carrier spacing can lead to exceeding the coherence interval in the frequency domain. It should be noted that all OFDM symbols have a short cyclic prefix, not only to reduce transmission overhead, but also because the large spacing between subcarriers means a small mean delay time [13].

The Orthogonal Time Frequency Space (OTFS) waveform was designed to ensure reliable communication in a strong Doppler environment. OTFS is a two-dimensional modulation technique that modulates data in the Doopler-delay (DD) domain, not in the time-frequency (TF) domain like in OFDM. This new modulation is very attractive for ISAC systems, as in the case of OTFS, both parts of the system (sensing/radar and communication) use the DD domain to represent the data [14]. More details on OTFS can be found in [15,16,17,18].

For the radar subsystem, there are two main approaches in (ISAC) system design, based on the estimation of the following: (1) the cross-ambiguity function (CAF) [19], and (2) the channel state information (CSI) [5]. The CAF-based processing practically does not depend on the waveform, and in the case of the OTFS, it could be the same as in the case of the OFDM [20]. However, the CSI-based approach extracts information about the radar target from the channel impulse response using pilots, so the pilot configuration in the case of CSI-based sensing plays an important role. A Zero-Padded OTFS (ZP-OTFS) [15,16] has the simplest pilot configuration among all OTFS waveforms. It has a dedicated pilot zone in the DD domain with only one active carrier in its center. Due to a low number of active carriers and a large number of inactive carriers, ZP-OTFS is not very efficient and its modification, ZP-BOX-OTFS, was introduced in [21]. The ZP-BOX-OTFS waveform has more active carriers in the center of the pilot zone of the DD grid, but is more complex from a computational point of view and still uses the guard interval (with zero values) in delay and Doppler directions.

In turn, a random padded OTFS (RP-OTFS), a hybrid method proposed recently, takes advantage of the OTFS technique for data and uses very short OFDM symbols for pilots [22,23,24,25,26]. The CSI estimation itself is performed in the TF domain, by means of these very short pilot-only OFDM symbols. Basically, RP-OTFS is a compromise between the strictly DD pilot configuration, such as ZP-OTFS, and OFDM. The advantages of RP-OTFS are the low computational complexity and the ability to use the well-known OFDM channel estimation approaches to OTFS after some adaptations. However, too high Doppler in the channel may corrupt the estimation results, as pilots in RP-OTFS are still OFDM symbols.

From a communication point of view, CSI estimation and bit detection are the most challenging parts of the processing, both for OTFS and OFDM waveforms. In the case of OTFS, additional difficulties arise when the reflection parameters (delay and Doppler offsets) are not consistent with the step size of the DD grid [27].

Reflection inconsistency with the DD grid could be in the delay direction, Doppler direction, or both at the same time. These effects are known as fractional delay, fractional Doppler frequency shift, or fractional reflection, respectively [28,29]. Creating a guard interval (i.e., cyclic prefix with random values) between the pilot and the data zone in RP-OTFS adds additional overhead, so in this paper, we examine the problem of interference between pilot carriers and data carriers. Also, because of a limited number of estimated samples of the CSI in the case of RP-OTFS (and also in ZP-OTFS and others), the CSI oscillations from the fractional reflections cannot be estimated directly. In addition, the accuracy of the CSI estimation is extremely important not only for communication but also, as mentioned before, for radar.

In the past few years, a substantial number of studies have been conducted on ISAC systems employing OTFS modulation. Several works have investigated the behavior of OTFS modulation in the presence of interferences and fractional reflections. The MIMO-OTFS sensing and communication model that takes into account both inter-symbol interference (ISI) and inter-carrier interference (ICI) is presented in [30]. In the radar part, the authors develop a generalized likelihood ratio test based multi-target detection and DD-angle estimation algorithm for MIMO-OTFS radar sensing that can simultaneously mitigate and exploit ISI and ICI effects. The authors in [31] propose a low-complexity impulse-based channel estimation algorithm for ZP-OTFS systems that effectively handles fractional reflections. Using the self-duality and invariance properties of a "pulsone" pilot (practically the ZP-OTFS pilot), the method achieves improved normalized mean squared error and bit error rate (BER) performance. The authors in [28] present the modified orthogonal matching pursuit algorithm [32] to estimate the delays and Doppler shifts that can be non-integer multiples of the system delay and Doppler resolutions, respectively. In [33] the authors propose a two-stage Bayesian learning-based approach for transceiver design and parameter estimation, which takes advantage of signal sparsity to improve ISAC system performance, and extend this to block sparse learning in mmWave ISAC systems.

In some articles [15,21], the authors argue that usage of the guard interval, consisting of inactive carriers in the DD domain, solves the problem of interference between data and pilot carriers, as in the case of ZP-OTFS or its ZP-BOX-OTFS modification. This is not completely true, especially in situations where the fractional reflection is close to the data carriers. An additional aggravating factor is that the power of the active pilot carriers in case of, for example, ZP-OTFS is concentrated in only one carrier, while in RP-OTFS, active pilot carriers are distributed over the whole pilot zone (in order to achieve the same power, the ZP-OTFS pilot carrier should be much stronger than in RP-OTFS). This means that the problems discussed in this paper are also applicable to other OTFS modifications.

The main contribution of this paper is the proposal of a method to reduce interference between pilot symbols and data in the ISAC system based on the RP-OTFS signal. This new approach leads to a significant decrease in BER during data transmission while preserving the high probability of the detection of moving objects, performed at the same time. In consequence, after the proposed enhancement, the application of the ISAC system based on the modified RP-OTFS is more beneficial than that of the OFDM-based and ZP-OTFS-based ones, as shown in the paper by simulation. Furthermore, in this work, a method is proposed for the simultaneous use of the DD grid by multiple devices in the presence of fractional delays, which is achieved by redistribution of interference power in the DD domain. Last but not least, the paper also provides insight into the selection of DD grid parameters for ISAC systems.

The structure of this article is as follows: In Section 2 the OTFS modulation and its different variants are presented and compared with OFDM. In Section 3, the random-padded OTFS modification is discussed in detail: its computational structure, the generation of random pilots, and the limitations of the RP-OTFS. Then, in Section 4, the system model for communication and radar subsystems is introduced. In turn, in Section 5 a new RP-OTFS version with reduced ICI, obtained by random pilot repetition, is proposed. Finally, in Section 6, some examples of the choice of DD grid parameters for the ICI-reduced RP-OTFS method are presented, and in Section 7, simulation results are shown that confirm the efficiency of the ICI-reduced RP-OTFS application compared with its predecessor, the regular RP-OTFS, as well as ZP-OTFS and OFDM. The paper ends with a discussion and conclusions.

## 2. The OTFS Waveform

In this section, the algorithmic basics of an OTFS waveform generation are presented only. Our goal is to provide the necessary background for the next part of the article. A detailed description of the OTFS modulation, with mathematical equations of definitions, features and relations, can be found in [14,15,21]. Here, as in our research, the application of the rectangular windowing is assumed only. For a better understanding of the OTFS, we will also describe the OFDM waveform generation here since in some of our simulations the OFDM performance is used as a baseline.

**Figure 1:**
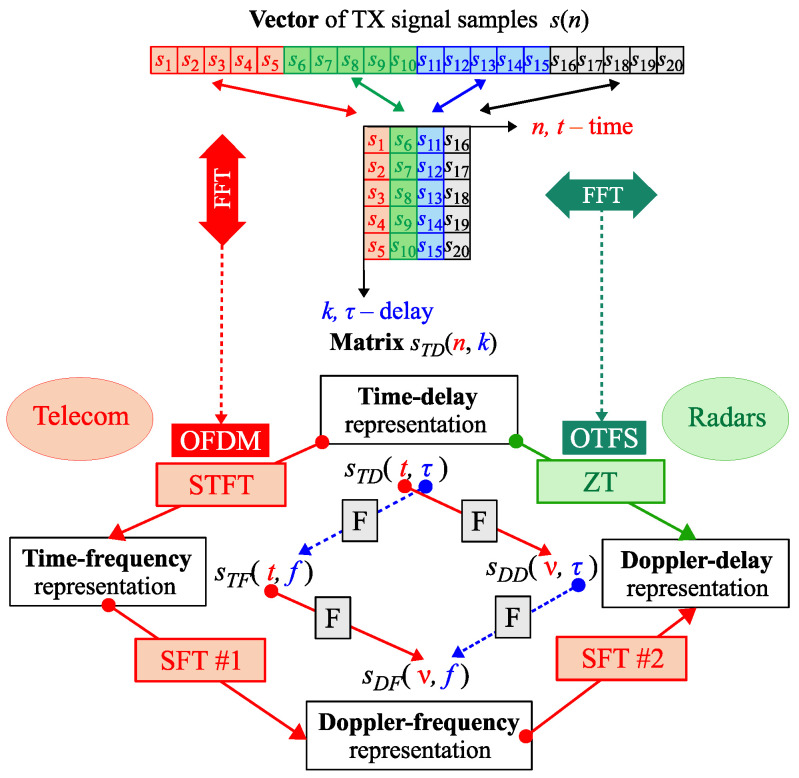
Graphical explanation of the relationship between OTFS and OFDM. Denotations: IR/FR-impulse/frequency response, F—Fourier transform, SFT—2D symplectic Fourier transform, STFT—short time Fourier transform, ZT—Zak transform.

In Figure 1 the time domain samples s(n) of a signal/waveform used in OTFS or OFDM are shown. The OTFS waveform is generated using the IQ data of the DD grid cells, while the OFDM waveform—the TF grid cells. Using a simple rearrangement, the signal/waveform s(n) can be represented in the time-delay (TD) domain as a complex-value matrix S with *M* rows (k=0,1,…,M−1) and *N* columns (n=0,1,…,N−1) (in some sources this matrix is called a long time–short time or slow time–fast time).

The DD and TD domains are connected with a pair of direct and inverse Zak transforms (ZT / IZT), more properties of ZT can be found in [15]. As shown in [16], the ZT (or IZT) of s(n) could be computed by applying the discrete Fourier transform (DFT) (or its inverse (IDFT)) to the rows of S. Applying DFT (IDFT) in the columns of S switches the signal between the TD and TF domains.

Concluding from Figure 1 and assuming the following relations: t↔n (time), τ↔k (delay), v↔m (Doppler) and f↔l (frequency), the generated and transmitted OTFS waveform $s_{TD}\left(n,k\right)$ can be computed in two ways from $s_{DD}\left(m,k\right)$ grid cells (i.e., from $s_{DD}\left(v,\tau \right)$):

1. directly via IZT, i.e., IDFT of $s_{DD}\left(m,k\right)$ rows:
  (1) $$\mathrm{DD}\to \mathrm{TD},\;\mathrm{IDFT}[\mathrm{rows}]:\;\;s_{TD}\left(n,k\right)=\frac{1}{\sqrt{N}}\sum_{m=0}^{N-1}s_{DD}\left(m,k\right)e^{j\frac{2\pi }{N}mn};$$
2. around via 2D inverse SFT of $s_{DD}\left(m,k\right)$ followed by IDFTs of columns of obtained $s_{TF}\left(m,k\right)$:
  (2) $$\begin{array}{cccc} \; & \mathrm{DD}\to \mathrm{DF},\;\mathrm{DFT}[\mathrm{cols}]:\;\; & \; & s_{DF}\left(m,l\right)=\frac{1}{\sqrt{M}}\sum_{l=0}^{M-1}s_{DD}\left(m,k\right)e^{-j\frac{2\pi }{M}kl}, \end{array}$$
  (3) $$\begin{array}{cccc} \; & \mathrm{DF}\to \mathrm{TF},\;\mathrm{IDFT}[\mathrm{rows}]:\;\; & \; & s_{TF}\left(n,l\right)=\frac{1}{\sqrt{N}}\sum_{m=0}^{N-1}s_{DF}\left(m,l\right)e^{j\frac{2\pi }{N}mn}, \end{array}$$
  (4) $$\begin{array}{cccc} \; & \mathrm{TF}\to \mathrm{TD},\;\mathrm{IDFT}[\mathrm{cols}]:\;\; & \; & s_{TD}\left(n,k\right)=\frac{1}{\sqrt{M}}\sum_{l=0}^{M-1}s_{TF}\left(n,l\right)e^{j\frac{2\pi }{M}kl}. \end{array}$$

It can be observed that the last step () in the second method is equivalent to the generation of the OFDM waveform $s_{TD}\left(n,k\right)$ from the given $s_{TF}\left(n,l\right)$ IQ values. Therefore, OTFS modulation can be interpreted as OFDM modulation with specific precoding. Consequently, the existing OFDM hardware can be used in joint OFDM and OTFS-based ISAC systems.

Here, it is important to note and remember that the transition from one domain to another can be accomplished by (I)DFTs in various ways (see the inner diamond in the lower part of Figure 1). When the original matrix (in the TD domain) contains the time-varying impulse response of the transmission channel in its columns, i.e., h(t,τ) replaces/exchanges s(t,τ), the DFTs of its rows give a Doppler-delay map of the transmission system (i.e., velocity-range map after appropriate scaling), while the DFTs of its columns result in a time-varying channel frequency response (CFR) of the system.

**Figure 2:**
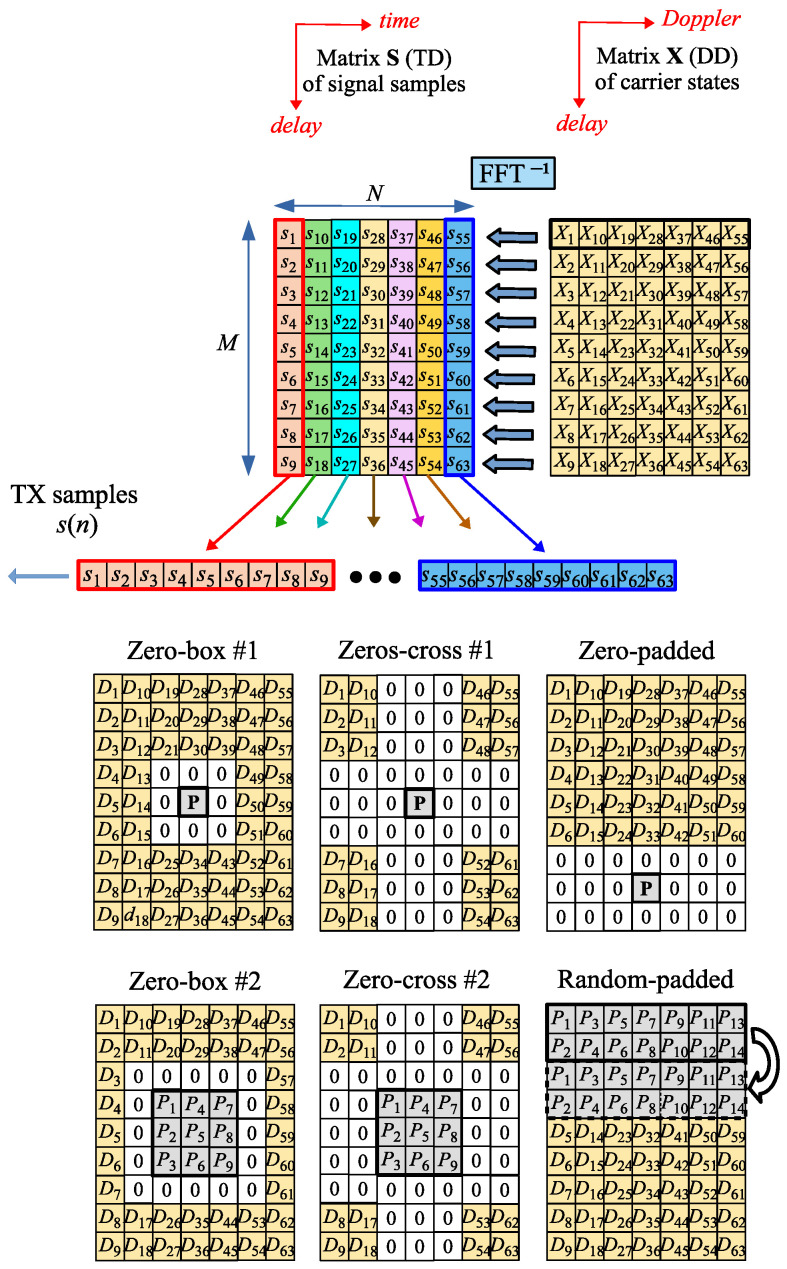
Principle of generation of the OTFS modulated waveform and different OTFS types.

The idea of OTFS modulation is explained separately with more details in the upper part of Figure 2, while in its lower part, different types of OTFS (different pilot vs. data arrangements) are compared. $s_{DD}\left(m,k\right)$, here and in the remainder of the paper, is noted as x(m,k)—transmitted IQ carriers (the received ones will be marked as y(m,k)). The complex value QAM (Quadrature Amplitude Modulation) symbols are entries in the DD matrix X. The inverse DFT is performed on its rows, i.e., along the Doppler direction, and then the TD matrix S is obtained. Then, the entries of the matrix S are transmitted column by column.

Like in the case of the OFDM waveform, OFTS needs to have pilots on the DD grid to estimate the CSI. The mutual arrangement of the pilots and the data can vary, but the DD grid is usually divided into two zones: the pilot zone and the data zone. In the lower part of Figure 2, different types of OTFS are compared with respect to different allocation strategies of pilot elements ($P_{k}$) and data elements ($D_{k}$) in the matrix X. $D_{k}$ are used to transmit data bits, while pilots ($P_{k}$) are used for OTFS frame synchronization and channel equalization.

The pilots do not necessarily occupy all subcarriers in the pilot zone, and some of the subcarriers can be inactive (the so-called guard carriers, which have a form of a strip, box, or cross). The guard interval is denoted by 0s in Figure 2. Also, depending on the configuration, the shape of the pilot zone and positions of the active (non-guard) carriers could be different. Guard carriers are used to reduce the interference between pilots and data carriers, but the drawback of many zero-value guard carriers is the reduction in pilot power. RP-OTFS [22,23,24,25,26] does not use guard carriers and offers better channel estimation characteristics. In this paper, RP-OTFS is discussed in its original form version [22,23,24,25,26] and in an improved form in the context of the ISAC system. The RP-OTFS represents a completely different approach in the OTFS design, since in its original proposition, the zero guard zone is missing and the pilots have randomly chosen values.

In the remaining part of the paper, we will use the following denotations: *M* is the number of rows of the X matrix (aka the number of elements in the delay direction of the DD grid), *N* is the number of columns of the X matrix (aka the number of elements in the Doppler direction of the DD grid).

## 3. The Random Padded OTFS

The RP-OTFS is an OTFS variant that is designed for a more efficient use of the resources of the DD grid—see Figure 2. In the case of RP-OTFS, the DD grid consists of two zones: the pilot zone and the data zone. The RP-OTFS grid structure is very similar to that of other OTFS variants. The main difference is the absence of the guard zone (inactive carriers with values set to zero) between the pilot zone and the data zone of the grid. These carriers in the RP-OTFS are used as additional carriers in the pilot zone.

The pilot zone of the RP-OTFS is 2L samples long in the delay direction and *N* in the Doppler direction of the DD grid. It is filled with very short OFDM symbols of length *L* (the length of the cyclic prefix (CP) is also *L*) that are used as pilots. They do not carry any data and they are filled only with pseudo-random patterns known to both the transmitter and the receiver.

### 3.1. RP-OTFS Transmitting and Receiving Process

**Figure 3:**
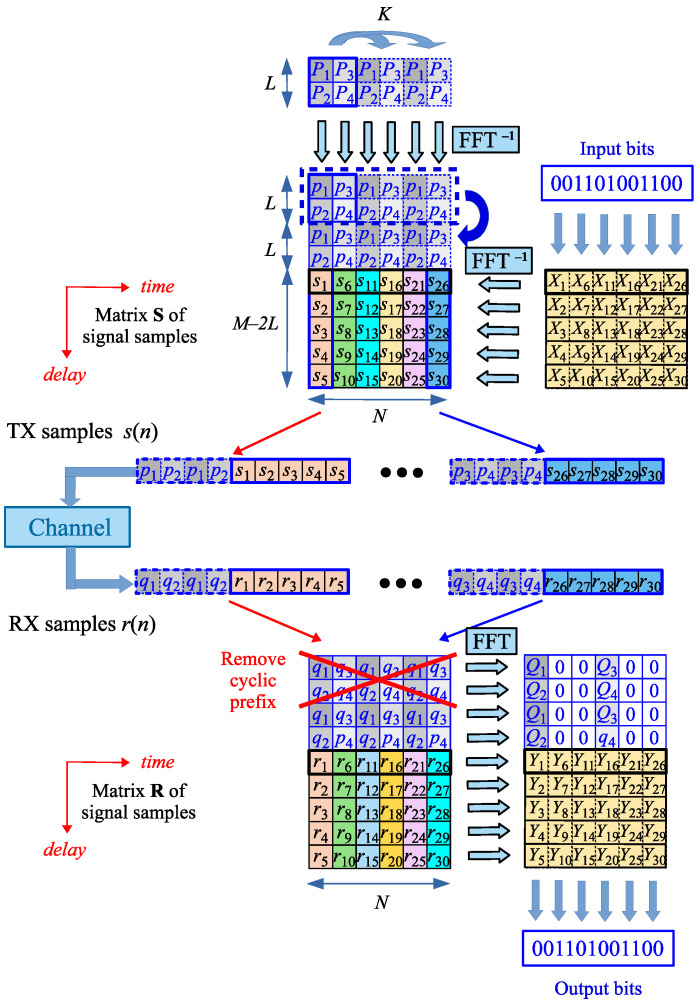
Principle of the RP-OTFS-based communication system.

The principle of the OTFS-based communication system with random padding was recently proposed [22,23,24,25,26], and is explained in Figure 3. The *K* parameter marked on the top of the picture and the repeating OFDM-pilots in the Doppler direction will be discussed in the next section.

In the RP-OTFS transmitter, the data part of the TD S matrix is obtained by the inverse Zak transform of the DD data matrix X that carries transmitted bits (computed as IDFTs of the rows of the matrix X—see Figure 2). However, in contrast to all known types of OTFS, the pilot samples $p_{k}$ are inserted directly into the matrix S with $s_{k}$ data in the TD domain as some of its additional first rows: an additional prefix. Then, samples of the “hybrid” TD-domain matrix S containing alternately blocks of pilot and data samples are transmitted column by column through a communication channel.

In the RP-OTFS receiver, all operations are performed in reverse order. First, the signal samples obtained ($q_{k}$—pilot, $r_{k}$—data) are put into columns of a receiver matrix in the TD domain. The matrix has dimensions M×N. Then, the cyclic prefixes of the OFDM-pilots are removed: the *L* first elements of each matrix column. The next elements *L* are used for the estimation of the instantaneous CIR, as explained in [26]. This is conducted using any ordinary OFDM CIR estimation technique [34].

The received samples $r_{k}$ create in the RP-OTFS receiver the matrix R with dimensions (M−2L)×N. The R contains only data samples in the TD domain. Pilot samples are used only for the estimation of the momentum CIR and are excluded from further processing dealing with bit decoding. Performing DFT on rows of R brings the signal back to the DD domain and, as a result, gives the matrix Y. The initial CSI estimation is conducted separately for each OFDM-like pilot symbol. At this stage, the instantaneous CSI estimations are acquired. Later, they are processed, and the final CSI estimate is obtained.

### 3.2. Generation of RP-OTFS Pilots

The generation of OFDM pilot symbols starts in TF domain. The pilot carriers are modulated using pseudorandom numbers $P_{k}$ originating from quadrature phase-shift keying (QPSK), i.e., $ℜ(P_{k})$ and $ℑ\left(P_{k}\right)\in \left\{-1,+1\right\}$. The real and imaginary parts of the modulating sequence are distributed uniformly; therefore, $ℜ(P_{k})$ and $ℑ(P_{k})$ are i.i.d. with the Radamacher distribution. After switching to TD or DD, the pilot samples will have an approximately normal distribution. The last statement is based on two facts:

– the discrete ZT is based on the DFT with some rearrangements;
– the DFT of the random variable has approximately normal distribution (Theorem 4.4.1 in [35]) with some limitations that are not applicable for our case.

The Radamacher distribution of complex pilot values $P_{k}$ in TF ensures a uniform energy distribution within OFDM-based pilot symbols [36]. This prevents the situation of having zero magnitude carriers in the OFDM-based pilot zone in TF, which causes division by zero in some types of channel equalizers, for example, in the least squares channel estimator (LS) [37].

After the generation of OFDM-based pilot symbols $P_{k}$ in TF, they are transformed into TD by IDFT/IFFT, values $p_{k}$ are obtained and CP is added. The CP length in the RP-OTFS is equal to the length of the OFDM symbol, that are used as pilot. The reason for this is that the pilot symbols do not contain any data carriers and are used only to estimate instantaneous CIR. This means that the maximum CIR length that can be estimated using an OFDM symbol is equal to the length of the symbol itself. As mentioned above, OFDM-based pilot symbols should not go through the Zak transformation from the DD domain to the TD or from the TD domain to the DD domain. They are directly placed on the TD grid (see Figure 3).

Some additional requirements regarding RP-OTFS pilots are discussed later in Section 3.3 on ICI between pilot and data carriers.

### 3.3. The RP-OTFS Application Limitations

The RP-OTFS pilots have the standard OFDM structure. The length of the OFDM pilot symbols, *L*, defines the maximum delay that can be estimated with its help, including fractional delay side lobes. However, since RP-OTFS is intended to work in a strong Doppler environment, the OFDM-based pilot will suffer from the violation of orthogonality between subcarriers. From this point of view, it is necessary to keep the pilot symbol as short as possible. The amount of distortion of the OFDM-based pilot symbol caused by the Doppler effect is called the carrier-to-interference ratio (C/I ratio). The interference coming to the *i*-th subcarrier from all other subcarriers (j≠i) inside one OFDM pilot symbol can be estimated using the following formula derived in [38]:

(5) $$CI\left(i\right)=\frac{1}{\frac{{\left(L/f_{s}\cdot f_{d}\right)}^{2}}{2}\sum_{\begin{array}{c} j=1\\ j\neq i \end{array}}^{L}\frac{1}{{\left(j-i\right)}^{2}}},$$

where $f_{d}$—the Doppler frequency shift, $f_{s}$—the sampling frequency.

**Figure 4:**
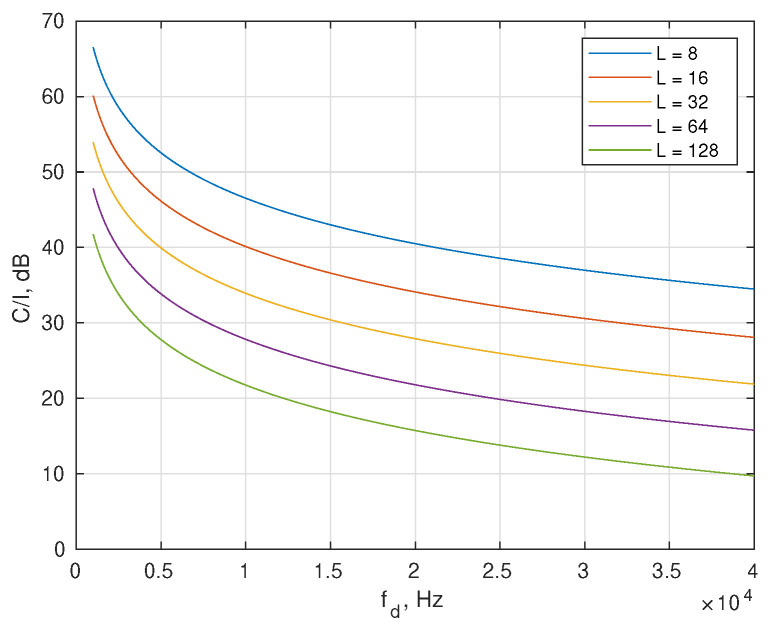
The C/I ratio as a function of Doppler frequency shift $f_{d}$ for different lengths of the pilot symbol *L*.

In Figure 4 the dependence of the C/I ratio on $f_{d}$ is shown for different lengths *L* of the pilot symbol. The carrier frequency in this example is 70 GHz. This frequency was chosen to be very close to the maximum carrier frequency (71 GHz) from the 5G specification [11]. It results from Figure 4 that RP-OTFS usage is possible in scenarios with a short impulse response. In Table 1 maximum velocities of moving objects are shown, for which the C/I ratio of the pilots is kept at least at the level of −40 dB and −50 dB.

**Figure 5:**
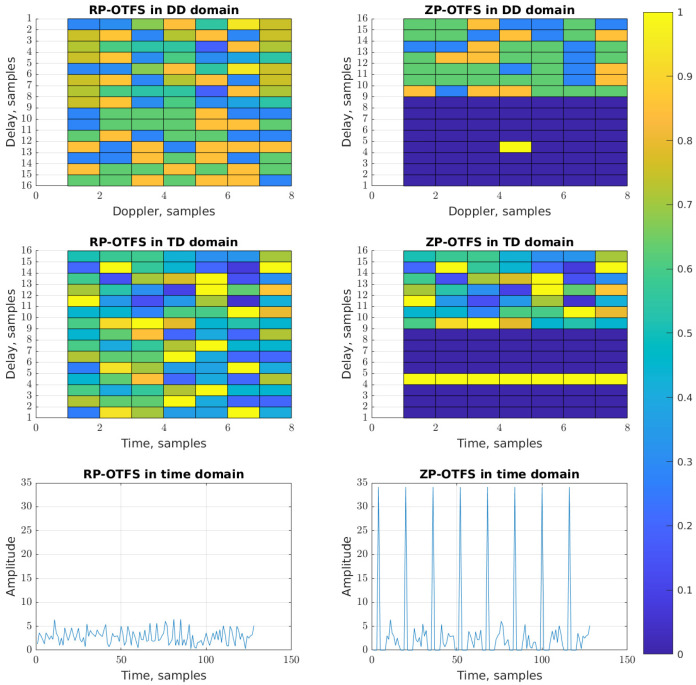
The RP-OTFS and ZP-OTFS waveforms in DD, TD and time domains.

It was mentioned in the introduction and also will be shown in the system model (Section 4) that in case of fractional reflections, the OTFS can lose its orthogonality and the carriers begin to interfere. This effect is also observed between the data carriers and between the pilot and the data carriers. In most modern communication standards, the pilot power is usually higher than the power of the data symbols [39,40]. This means that even stronger interference can be expected between pilots and data carriers. However, unlike the ZP-OTFS, where the whole power of the pilot zone is concentrated in only one active pilot carrier, the RP-OTFS is devoid of this drawback because it distributes the total energy among a large number of active pilot carriers. With the same total power in the pilot zone as in ZP-OTFS, the power of each active pilot carrier of the RP-OTFS will be ${\left(NL\right)}^{2}$ times lower. This makes RP-OTFS attractive from the ICI reduction and Peak-to-Average Power Ratio points of view. In Figure 5 an exemplary RP-OTFS and ZP-OTFS in the DD, TD, and time domains are depicted. The power of the pilots for both waveforms is the same.

Consequently, the OTFS pilot pattern is optimized for channels characterized by moderate Doppler shifts and short delay spreads. The dispersion of pilot energy across multiple subcarriers facilitates the detection of targets with lower radar cross sections (RCS), as will be demonstrated in the simulation section. One representative example of such a channel is an automotive ISAC scenario [41].

## 4. The System Model

### 4.1. The Communication System Model

The considerations presented in this section are based on [15,16].

The OTFS waveform uses the DD domain to modulate the IQ data. It is important to note that below, the exchange of meaning between variables *m* and *k* is made compared with Section 2, where *k* denotes the delay and *m* the Doppler frequency shift. Currently, the notation is reversed. As a consequence, Doppler-delay matrices are now considered as delay-Doppler, as in [15]. This is a result of the inconsistency in the historically established names of the domains: time-frequency and Doppler delay. The time dimension in TF (or TD) results in the Doppler dimension in the Doppler-delay representation. However, the term “delay-Doppler” is commonly used in modern literature.

Let us assume that on the DD grid on the transmitter side, we have $X\in C^{M\times N}$, where *M* is the number of carriers in the delay direction and *N* is the number of carriers in the Doppler directions. Let the signal sampling frequency be $f_{s}$. Now we can calculate basic signal and resolution parameters, as well as the maximum delay and maximum Doppler of the DD-grid. The length of the OTFS frame is $T=M\cdot N/f_{s}$. We assume that the OTFS signal is critically sampled and, therefore, the bandwidth of the signal $B=f_{s}$. The delay resolution, Doppler resolution, maximum delay, and maximum Doppler are equal to $1/f_{s}$, $f_{s}/M$, $M/f_{s}$, and $N\cdot f_{s}/M$, respectively. Transformation from DD to TD domain is performed using the inverse DFT over DD matrix rows (operation known as the inverse Zak transform), while transformation from TD to DD domain—using the DFT over TD matrix rows (operation known as the Zak transform) [16].

Let the received signal be denoted on the DD grid as $Y\in C^{M\times N}$. The vertical vectors $x_{m}={\left[X\left(m,0\right),\;X\left(m,1\right),\ldots ,X\left(m,N-1\right)\right]}^{T}$ and $y_{m}={\left[Y\left(m,0\right),\;Y\left(m,1\right),\ldots ,Y\left(m,N-1\right)\right]}^{T}$ are the *m*-th rows of X and Y, respectively: $x_{m},\;y_{m}\in C^{N\times 1}$. By combining vectors $x_{m}$ we define the vertical vector $x\in C^{MN\times 1},\;x={\left[x_{0},x_{1},...,x_{M-1}\right]}^{T}$, similarly to $y\in C^{MN\times 1},\;y={\left[y_{0},y_{1},\ldots ,y_{M-1}\right]}^{T}$. For simplicity, we will normalize the delay and Doppler to the DD grid resolution in the following way: $\ell_{i}=\tau_{i}\cdot \frac{T}{MN}$ is a normalized delay, and $\kappa_{i}=\frac{\nu_{i}}{NT}$ is a normalized Doppler. Here, it is important to note that $\ell_{i}$ and $\kappa_{i}$ are not necessarily integers; they could be fractional values, as mentioned in the Introduction. We will denote the set of all time delays by $L=\{\ell_{i}\}$, and the set of all Doppler frequency shifts by $K_{\ell }=\left\{\kappa_{i}\;|\;\ell =\ell_{i}\right\}$. The complex gain/attenuation, corresponding to the delay $\ell_{i}$ and the Doppler offset $\kappa_{i}$, is denoted as $\nu_{\ell }\left(\kappa \right)$. At this stage, we assume that the maximum delay of reflections is not very large (a few samples). We also assume that the Nyquist sampling theorem is also met in the Doppler direction, that is, $-N/2\leq \kappa_{i}\leq N/2$.

It is shown in [16], that in the general case of fractional delay and fractional Doppler, the CSI can be expressed in the DD domain by the following formula:

(6) $$\nu_{m,l}\left(k\right)=\frac{1}{\sqrt{N}}\sum_{\ell \in L}\left(\sum_{\kappa \in K_{\ell }}\nu_{\ell }\left(\kappa \right)\underset{Doppler\;component}{\underset{︸}{e^{j2\pi k(m-l)}\zeta_{N}\left(\kappa -k\right)}}\right)\underset{Delay\;component}{\underset{︸}{\mathrm{sinc}(l-\ell ),}}$$

where $\zeta_{N}\left(x\right)=\frac{1}{\sqrt{N}}\frac{\sin(\pi x)}{\sin(\frac{\pi x}{N})}e^{\frac{j\pi x(N-1)}{N}}$ is a periodic sinc function, *m* and *k* are the sample coordinates on the DD grid in the delay and Doppler directions, respectively, m∈0⋯M−1,k∈0⋯N−1. *l* is a time shift of the CSI. We assume that CSI changes in time due to the Doppler effect: l∈0⋯(M−m)N, m,k,l∈Z. The resulting signal, after passing the Doppler multipath wireless channel, is equal to the following summation of circular convolutions (for each delay) in the DD domain [16]:

(7) $$y_{m}=\sum_{l\in \ell }\nu_{m,l}⊛x_{m-l}.$$

**Figure 6:**
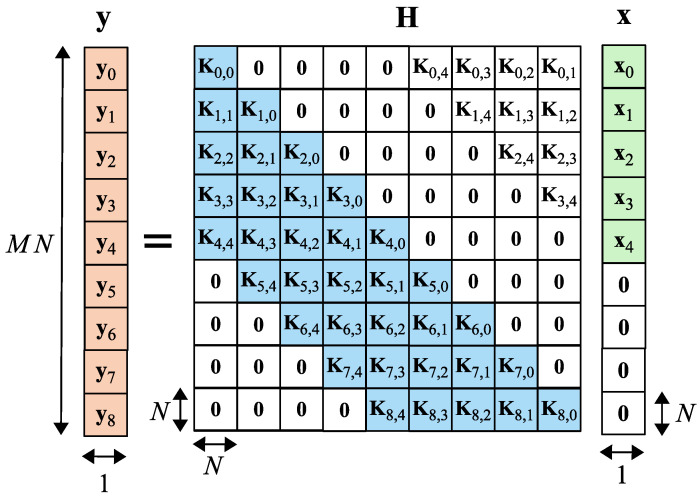
Input-output relation for OTFS modulation with zero padding in the DD domain [16].

The resulting CSI matrix H consists of the submatrices $K_{m,l}\in C^{N\times N}$. Each of them is defined as (see Figure 6) [16]:

(8) $$K_{m,l}=\left[\begin{array}{cccc} \nu_{m,l}\left(0\right) & \nu_{m,l}\left(N-1\right) & \ldots & \nu_{m,l}\left(1\right)\\ \nu_{m,l}\left(1\right) & \nu_{m,l}\left(0\right) & \ldots & \nu_{m,l}\left(2\right)\\ \vdots & \vdots & \ddots & \vdots \\ \nu_{m,l}\left(N-1\right) & \nu_{m,l}\left(N-2\right) & \ldots & \nu_{m,l}\left(0\right) \end{array}\right].$$

The arrangement of the submatrices $K_{m,l}$ (8) inside the matrix H is as follows:

(9) $$\begin{array}{cc} H[\;mN+1\ldots (m+1)N,\;(m-l)N+1\ldots (m-l+1)N\;] & =\\ =\left\{\begin{array}{cc} K_{m,l}, & m\in 0\cdots M-1,\;\;l\in 0\cdots (M-m)N,\\ 0, & \mathrm{otherwise}. \end{array}\right. \end{array}$$

The visualization of the placement of the submatrices $K_{m,l}$ within the matrix H is given in Figure 6.

Here we want to draw the Readers’ attention to two important details about H: the matrix H describes the CSI directly in the DD domain and the format of the matrix H is uncommon. The change in the format of the matrix H is due to the fact that the OTFS symbol has two dimensions (2D), unlike OFDM (1D).

Based on the above description, the signal that passes through the multipath channel with Doppler frequency shifts can be written as [16]:

(10) y=H·x+w,

where w is a noise vector in the DD domain.

The vector w is defined as $w_{m}={\left[W\left(m,0\right),W\left(m,1\right),\ldots ,W\left(m,N-1\right)\right]}^{T}$, $w={\left[\;w_{0},\;w_{1},\;\ldots ,w_{M-1}\;\right]}^{T}.$ The W matrix is the result of the Zak transform performed on the time domain noise vector $w∼N(0,\sigma^{2})$, where $\sigma^{2}$ is the variance of the noise (here we assume that the rectangle windowing function was used in the Zak transform). After the Zak transform of *w*, one obtains $w∼N(0,\sigma^{2}/N)$ [42].

Based on the above description, the signal that passes through the multipath channel with Doppler frequency shift can be modeled directly in the DD domain using the Bayesian General Linear Model [43]. This allows us to directly use the well-known estimation and bit detection methods designed/developed for OTFS signals, for example, the linear minimum mean square error (LMMSE). The LMMSE estimate of the transmitted data $d_{k}$ is defined as [15]:

(11) $$\hat{d}={\left[F_{N}⊗I_{M}\right]}^{†}H^{†}{\left[HH^{†}+\frac{\sigma_{w}^{2}}{\sigma_{d}^{2}}I\right]}^{-1}y,$$

where $F_{N}$ is the discrete Fourier transform matrix of size *N*, $I_{M}$ is the identity matrix of size *M*, ⊗ is the Kronecker product, † denotes complex conjugation and transposition, and finally, $\sigma_{w}^{2}$ and $\sigma_{d}^{2}$ are noise and data variances (see Figure 6). The fast algorithm for solving (11) is proposed in [44]. The whole channel estimation and equalization process can be performed in the DD domain. In this work, the Matlab code of the LMMSE equalizer [15], designed for ZP-OTFS, is used.

### 4.2. The Radar System Model

The radar channel model is based on the communication model (10). Suppose that the radar channel has $\tilde{L}=\tilde{\ell_{i}}$ delays and the set of all Doppler frequency shifts $\tilde{K_{\ell }}=\left\{\tilde{\kappa_{i}}\;|\;\tilde{\ell }=\tilde{\ell_{i}}\right\}$. Then similarly to (6), we can rewrite

(12) $${\tilde{\nu }}_{m,l}\left(k\right)=\frac{1}{\sqrt{N}}\sum_{\tilde{\ell }\in \tilde{L}}\left(\sum_{\tilde{\tilde{\kappa }}\in \tilde{K_{\ell }}}{\tilde{\nu }}_{\tilde{\ell }}\left(\tilde{\kappa }\right)e^{j2\pi k(m-l)}\zeta_{N}\left(\tilde{\kappa }-k\right)\right)\mathrm{sinc}\left(l-\tilde{\ell }\right),$$

where ${\tilde{\nu }}_{\tilde{\ell }}\left(\tilde{\kappa }\right)$ is the RCS of the target with delay $\tilde{\ell }$ and $\tilde{\kappa }$ Doppler shift.

By applying transformations analogous to those used in the communication model, the final radar channel model is derived:

(13) $$\tilde{y}=\tilde{H}\cdot x+w.$$

In this article, we focus only on the CSI-based approach, explained in [23,26], as it has a more common processing part with the communication subsystem than CAF. In the CSI-based approach, the results of the OFDM-like pilot-based CIR estimation are used to detect the targets. This reduces the complexity of radar processing significantly lower than in the case of CAF-based processing. Another not-so-straightforward disadvantage of the CAF is that the limited freedom of choice of waveform parameters can be critical for the shape of the ambiguity function. This limitation comes from the fact that the waveform structure we choose should allow the decoding of the transmitted data in a receiver. For this reason, some additional communication components should be inserted into the transmitted signal that are treated as unwanted “artifacts” by the radar subsystem, e.g., repeating reference pilot signals and repeating fragments of OFDM waveforms called a cyclic prefix. The repeatable parts of the signal generate undesirable peaks in CAF, so-called ghosts [25].

In the CSI-based approach, the processing begins with the $\tilde{H}$ matrix estimation. The channel matrix estimation step is common for both subsystems: communication and radar. The process of the CSI estimation in case of RP-OTFS is described in [26].

Each subdiagonal of the $\tilde{H}$ contains information about specific $\tilde{\ell }$, $\tilde{\kappa }$ and ${\tilde{\nu }}_{\tilde{\ell }}\left(\tilde{\kappa }\right)$. This way of presenting information is not suitable for standard radar processing algorithms. Therefore, before target detection, an additional step is needed to transform $\tilde{H}$ to the DD grid (different delays in rows, different Doppler frequency shifts in columns). The transformation should be performed as follows:

(14) $${\tilde{H}}_{DD}\left[m,k\right]=\tilde{H}\left[m\cdot N+k,l_{0}\cdot N\right],$$

where $l_{0}$ is the time shift of the CSI estimation relative to the beginning of the OTFS frame. In most practical cases $l_{0}=0$, the range of $l_{0}$ is 0…L−1.

**Figure 7:**
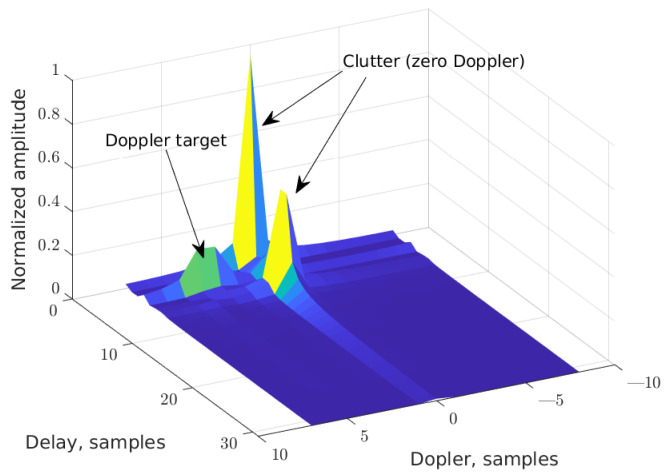
Corresponding DD matrix obtained from estimated exemplary **H** matrix.

In Figure 7 an exemplary result of the matrix ${\tilde{H}}_{\mathrm{DD}}$ conversion is shown. Reflections from two static objects (so-called clutter) and from one moving object/target are visible in it.

The obtained DD matrix should be processed by any standard radar detection algorithm, such as the constant false alarm rate (CFAR) [19]. The detection of targets performed by the radar subsystem is beyond the scope of this paper. However, in order to compare the performance of the RP-OTFS in simulations with other waveforms, we have estimated the processing gain for different modulation schemes. This gain was defined as a ratio of the output SNR (defined as the power of the DD cell with the target and noise, after radar processing, divided by the power of the DD cells with noise only) to the input SNR (signal-to-noise ratio at the input of the receiver) and was calculated for different input SNR values.

### 4.3. CIR Estimation

Analyzing the instantaneous CIR estimation processes in the case of OFDM and OTFS, there are some common points. The main peculiarity is that in the case of OFDM, the data and the pilots are both inside an OFDM symbol. In contrast, in the case of the RP-OTFS, the data are located separately between OFDM-based pilots.

After obtaining the estimation of the instantaneous CIR for each delay in the range of [0…L] samples, the sample in position n=L+l+iM, i∈{0,…,N−1}, l∈{0…L} contains instantaneous CIR information at the *l* samples delay. In the RP-OTFS, we obtain the CIR estimate for the delay *l* once per *M* samples. The complete CIR can be recovered by interpolating the instantaneous CIR estimations for each *l* separately (in OFDM, the CFR estimate is interpolated).

The choice of the interpolation method is based on the expected behavior of the CIR function, mainly on its derivatives. We will base our interpolation analysis on (6), which takes into account the effect of fractional reflections. But during analysis of the CIR function, we do not consider the particular DD grid; therefore, we can assume that no fractional reflection occurs. Then we can also assume that (κ−k)and(l−ℓ)∈Z, hence $\zeta_{N}\left(\kappa -k\right)=\sqrt{N},\sin c\left(l-\ell \right)=0$ and

(15) $$\nu_{m,l}\left(k\right)=\left\{\begin{array}{c} \nu_{\ell }\left(\kappa \right)e^{2\pi j\kappa (m-l)/MN},\;\;\;\mathrm{if}\;l=\ell \;\mathrm{and}\;k={\left[\kappa \right]}_{N},\\ 0,\;\;\;\mathrm{otherwise}.\; \end{array}\right.$$

By calculating the derivatives of (15) over *m*, one obtains the following recurrent formula:

(16) $$\frac{\partial^{p}\;\nu_{m,l}\left(k\right)}{\partial \;m^{p}}={\left(2\pi j\kappa /MN\right)}^{p}e^{2\pi j\kappa (m-l)/MN}.$$

As $\lim_{p\to \infty }{\left(2\pi j\kappa /MN\right)}^{p}=0$ for MN>>κ, the magnitude of the derivatives will decrease with increasing *p*. The rate of decrease in derivatives depends on the size of the grid MN and on the Doppler frequency shift κ. This means that for the same grid size, the optimal interpolation method depends on the environment. For the cases where the Doppler effect is not very high, low-order interpolating polynomials can be used, for example, simple linear interpolation. At the same time, when the value of κ increases, more advanced interpolation methods, such as cubic spline interpolation, should be exploited. Generalizing to the more general case: when the Doppler frequency shift is not constant during the RP-OTFS frame, for example, when a target is accelerating, analysis of Equations (15) and (16) is more complicated. In this case κ becomes dependent on *m* and additional degrees of freedom of an interpolation function are required to fit it.

After obtaining CSI estimation in the time domain (i.e., CIR $\nu_{m,l}\left(k\right)$), Equations (6)–(9) are used to transform CSI to the DD domain and model data transmission directly in the DD domain with (10).

**Figure 8:**
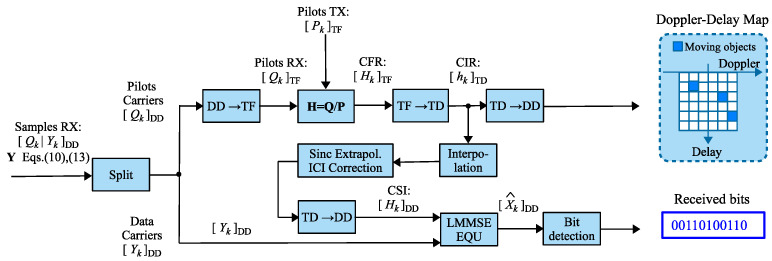
The system model based processing diagram.

Based on the system model described above the block diagram of the receiver processing is shown in Figure 8. On the receiver side, it is assumed that the signal is perfectly synchronized with the strongest path of the multipath environment. It is also assumed that the signal is already transformed to the DD domain, so the processing is conducted mainly in the DD domain, except for the channel estimation part, because of the nature of OFDM-based pilots. The subsequent processing is divided into two branches: radar system processing and communication system processing.

In the radar subsystem, only OFDM pilots are processed; the rest of the DD grid is ignored. First, the pilots are transformed to the TF domain where the instantaneous CFRs are estimated for each OFDM pilot separately using the Least Squares method. The CFR estimates are then transformed to the TD to obtain an instantaneous CIR. The CIR estimates are used in the communication processing branch. Finally, a total of *N* CIR estimates is used to construct the DD map for the radar detector.

The results of the channel estimation are also utilized in the second branch—the communication processing path. Based on instantaneous CIR estimates, interpolation and compensation for ISI caused by fractional reflections (will be discussed in Section 5.2) are performed. The resulting CSI, after being transformed into the DD domain, is passed to an equalizer employing the LMMSE algorithm. The equalized signal in the DD domain is then forwarded to the bit detection block, which outputs the demodulated bits of the transmitted message.

## 5. Interference Reduction

If *ℓ* or/and κ are not integers, i.e., the given propagation path (or target in radar case) contains fractional reflection components, then it follows from Equation (6) (or (12) in radar case) that such reflections generate oscillations in both delay (sinc component in the equations) and Doppler ($\zeta_{N}$ component in the equations) directions. The oscillating components propagate throughout the whole DD grid and cannot be directly estimated by RP-OTFS’ pilots. In this section, we will discuss methods of reducing this effect.

### 5.1. Proposed Pilot-to-Data Interference Reduction

Here we will discuss the interference between the pilot zone and the data zone and the ways to reduce it. Usually, the biggest part of reflections in a multi-path environment comes from the objects that have similar Doppler frequency shift, for example, from huge static objects like buildings in the cities. Eliminating the influence of these reflections could significantly reduce the level of ICI between pilots and data. The method introduced here is based on DFT properties for periodic signals [45]. If a signal consists of K∈N repetitions of some basis *P* samples long sequence z(p),p=0…P−1, that is

(17) $$\tilde{z}\left(n\right)=\left\{\underset{z(p)}{\underset{︸}{z(0),z(1),\ldots ,z(P-1)}},\underset{z(p)}{\underset{︸}{z(0),z(1),\ldots ,z(P-1)}},\ldots \right\},$$

and if $\tilde{z}\left(n\right)$ has zero mean, then the DFT of $\tilde{z}\left(n\right)$, i.e., ${\tilde{Z}}_{k}$, will be equal to the K−1 times up-sampled the DFT of z(n), i.e., $Z_{k}$:

(18) $${\tilde{Z}}_{k}=\left\{\begin{array}{cc} Z_{k}, & k\in \{\;0,\;K,\;2K,\ldots ,PK\;\},\\ 0, & \mathrm{otherwise}. \end{array}\right.$$

In fact, this effect has already been presented in Figure 3: it was assumed there that the pilot samples repeat K=3 times and, as a result, K−1=2 zeros are present between non-zero values of the pilot DFTs in the resulting matrix.

On the receiver’s side, in case of a fractional delay, the pilot of the RP-OTFS will interfere with the data. In case of pilot repetition in the time (slow-time, long-time) direction in the TD domain (corresponds to Doppler direction in the DD domain), the interference signal will also repeat. Hence, using the above property, the ICI energy distribution on the DD grid could be controlled. To ensure that the number of repetitions remains an integer within a single RP-OTFS frame, the condition PK=N must be satisfied.

**Figure 9:**
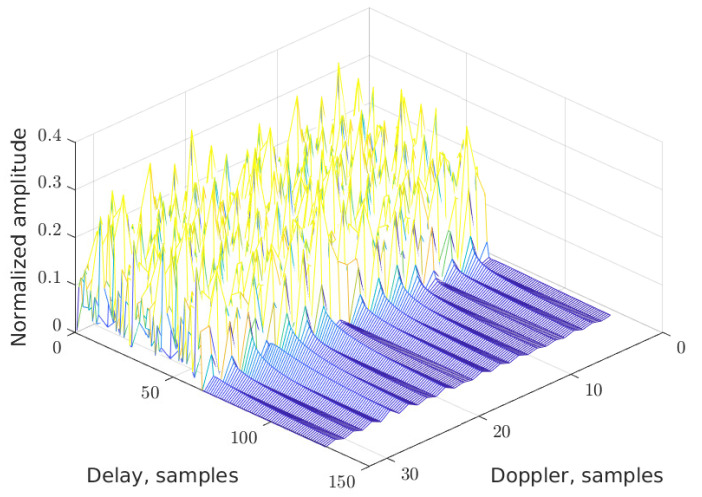
The DD map for RP-OTFS with $K=\frac{N}{2}$.

**Figure 10:**
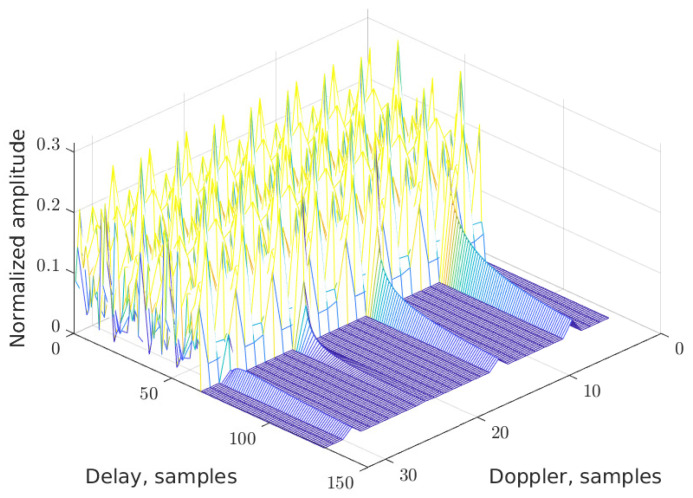
The DD map for RP-OTFS with $K=\frac{N}{8}$.

**Figure 11:**
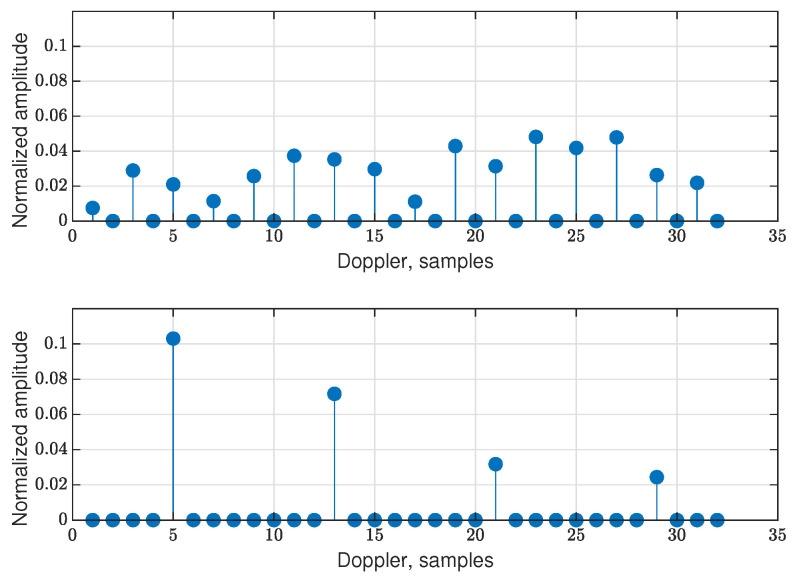
The cut of the DD grid at the beginning of the data zone (filled with zeros) along the Doppler axis for the RP-OTFS with $K=\frac{N}{2}$ (up) and $K=\frac{N}{8}$ (down).

As an example, the DD grid without data (that is, for pilots only) is shown for the new RP-OTFS in two figures: for $K=\frac{N}{2}$ in Figure 9 and for $K=\frac{N}{8}$ in Figure 10. In both cases, the total power of the pilots is the same, and fractional signal reflections are present. It is clearly visible that interference from pilot to data zone is distributed in a completely different manner: in the first case, it is smeared equally in the data zone, while in the second case, it is concentrated locally only in several stripes (see Figure 11).

The columns in the DD grid, where the ICI will be concentrated, could be chosen by multiplying the rows of the TD matrix (only the part of the matrix that contains the pilot samples) by $e^{2\pi ki/N}$, i=0,1,…,N−1, k∈0…K−1. This will shift the ICI stripes in the Doppler direction by the *i* columns of the DD grid and gives additional flexibility in controlling the positions of the reflections. Only the following columns of the DD grid will be corrupted by ICI :

(19) n=i+Kj,j∈{0,1,…,N/K}.

It should be noted that the number of repetitions can be equal to any integer from 0…N. The boundary case, where K=N, means that all pilot symbols are the same. In this case, all ICIs from objects with the same Doppler will be concentrated in one single column. On the other hand, case K=0 means that all OFDM pilots are different. In this situation, all ICIs from the objects are uniformly spread across all carriers of the DD grid.

By choosing the parameters *K* and *i*, the ICI caused by the pilots can be adapted to different scenarios. For example, K=0 could be useful when there is no expectation of strong reflections with fractional delays. In cases when the amount of data to transmit is not very high, some of the carriers of the DD grid could be excluded from data transmission. It makes sense to use these data-free columns to concentrate ICI on them. This can be conducted by increasing *K* and choosing appropriate *i* if the nonzero pilot slices, present in the DD grid, should be shifted.

**Figure 12:**
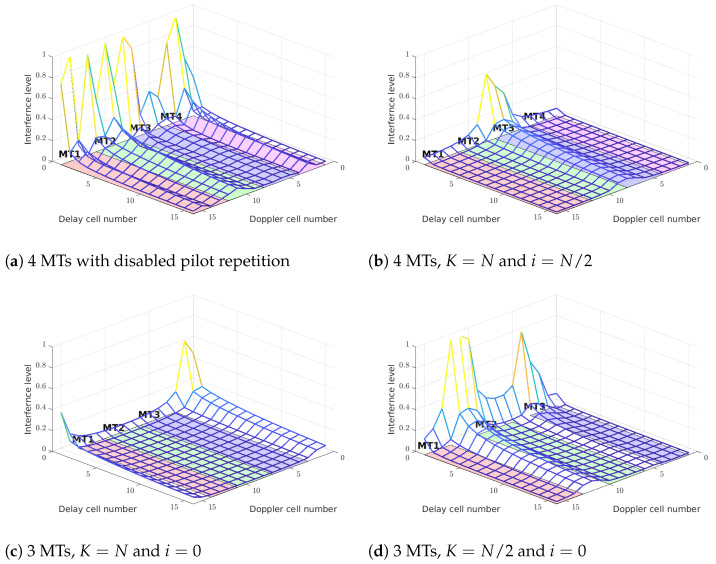
Different scheduling MT scenarios with different pilot repetition parameters, but the same channel. Without the pilot repeating feature, the interferences form pilots has the highest level (**a**). Only MT-2 and MT-3 have significant interferences (**b**). In case of 3 MTs, two options could be used to reduce interference between (**c**,**d**). The interferences are combined incoherently.

The pilot repetition is particularly important in scenarios where multiple mobile terminals (MTs) transmit data over the same DD grid, by sharing different parts of the grid, e.g., different columns of the DD matrix. Each MT uses its own pilots and data symbols, while the data occupy only a portion of the grid (as illustrated in Figure 12). Since, in general, MTs are located in different positions, each of them experiences a distinct channel characterized by unique delays and Doppler shifts. As discussed previously, a mismatch between the channel and the grid causes oscillations.

If, for instance, the channel of one MT (1) is not aligned with the grid of another MT (2), pilots of MT-1 can induce oscillations within the grid region of MT-2. Repetition of the pilots redistributes the power of the interference and allows these oscillations to be concentrated in specific regions of the grid while suppressing them in some other, more critical regions. Moreover, due to the random phase of the complex channel coefficients, the resulting interference adds up incoherently, which further improves the signal-to-interference power ratio. This makes multiple MT transmissions more reliable.

The figure below illustrates the case of interference between MT-1 and three other mobile terminals (MT-2, MT-3, MT-4), where the same channel is used in all cases. It can be seen that the use of repeating pilot patterns significantly reduces the level of interference.

### 5.2. Proposed Data-to-Data Interference Reduction

The data-to-data interference reduction is based on extrapolating the CSI. The delay component in (6) is represented by the function sinc and the Doppler component does not depend on the fractional part of the delay. This means that any fractional delay will produce sinc oscillations in the CIR, which will generate interference not only between pilots and data, but also between data carriers. Since the RP-OTFS pilot zone occupies all columns in the Doppler direction for 2L pilot delays, and based on (6), we treat each particular column of the received DD grid as a separate one in the context of this algorithm. We also assume that the maximum reflection delay is L−2, which is equivalent to the statement that there is no reflection, at least in the last two samples of the CIR.

Based on the assumptions above, we can reconstruct the side lobe generating factor using the following formula, which was found experimentally:

(20) F(q)=A·sinc(0.5+q),

where *q* is the sample number (q=0…M−L−1) and *A* is a complex parameter that should be estimated. After receiving and estimating the channel (this process is described in the previous Section 4.3 on CIR estimation), we use the last two samples of CIR to estimate the complex parameter *A*. Estimation of *A* is conducted separately for each column of the DD grid.

The maximum delay, which could be estimated in **H** in case of RP-OTFS, is *L*. We will denote this estimate of part of **H** as **H**_*L*_. Then we append the reconstructed F(q) to **H**_*L*_ and obtain the reconstructed CIR matrix. Practically, we extrapolate the estimated part of the **H** matrix with the sinc function with the estimated parameter *A*.

To check the efficiency of the data interference compensation method based on the sinc extrapolation, proposed above, we provide the results in the simulation Section 7.

## 6. Choosing DD Grid Parameters for Different Use Cases

In this section, we will present three examples of choosing values of the global parameters (*M*, *N* and *L*) of the DD grid in the radar context. We start a discussion of each example with an introduction of its use case scenario, and then present an adaptation of the grid parameter values. We define three use-cases for ISAC:

A. very high speed: civil airplanes, high speed trains, etc; in this group, we consider flying objects with speed *v* in the range 120…340 m/s;
B. medium speed: vehicular, drones; in this group, we have moving objects with speed *v* in the range 10…60 m/s;
C. very low speed: pedestrians, low speed vernaculars; in this group, we have objects moving with speed *v* up to 2 m/s.

As the Doppler frequency shift $f_{d}$ depends not only on the movement velocity *v* of the objects but also on the carrier frequency $f_{c}$, as an example, we choose the carrier frequency to be the same as in the previous section, i.e., equal to 70 GHz:

(21) $$f_{d}=\frac{v}{\lambda }=\frac{vf_{c}}{c}.$$

where $\lambda =\frac{c}{f_{c}}$ denotes a wavelength and *c* is the speed of light. For unambiguous Doppler measurements, the sampling rate in the Doppler direction should satisfy the Nyquist theorem, and therefore, should be defined as $f_{s}^{\left(Doppler\right)}=\frac{f_{s}}{M}$. Using Equation (21), the maximum value of the parameter *M* that meets this requirement is given by

(22) $$M_{max}=\frac{f_{s}}{2\cdot f_{d}}.$$

Next, we take into account the range resolution of the DD grid. Communication systems operating in the range of tens of gigahertz typically make use of the bandwidth (BW) of tens or even hundreds of megahertz. As a sampling rate $f_{s}=BW$ of the complex signal, this gives a very high range resolution. For example, $BW=f_{s}=100$ MHz gives a resolution of up to 3 m. Such a high resolution will be useless for use-case A, but could be useful for use-case B. The same time resolution of 3 m may not be enough for some applications with targets from use-case C. In such cases, an increase in the BW should be considered.

Another aspect that should be taken into consideration for ISAC systems in the context of their radar sections is the value of integration time, that is, how long targets from different use cases (A, B, or C) can stay in one resolution element of the DD grid. For resolution in range direction, the calculations are trivial: the time required for a moving object to cross the resolution element is $\Delta t_{element}=\frac{c}{f_{s}\cdot v}$. In order to be able to combine the reflection coherently during the Zak transform, the frame time duration should be much shorter than the time spent by the target in one resolution element [19]:

(23) $$\frac{NM}{f_{s}}\ll \Delta t_{element}.$$

By changing the parameters of the DD grid *M* and *N* (keeping their product MN constant), different resolutions in Doppler and maximum unambiguous Doppler can be achieved. In terms of computational complexity, decreasing *N* (and increasing *M*) is more efficient than decreasing *M* (and increasing *N*). The computational complexity of the discrete Zak transform is O(MNlogN). In terms of RP-OTFS, keeping *N* as small as possible is also more profitable because the pilot overhead of RP-OTFS is directly proportional to *N*. *N* defines the number of OFDM pilots. On the other hand, the decrease in the value of *N* will result in worse Doppler resolution.

Below is an example of how to choose the RP-OTFS parameter values in use case B. At the end of this part, for illustration purposes, we provide a general table with results for the three use cases. Before we start, it is important to note that applying the RP-OTFS to targets from use case A is possible in an environment where all propagation paths have practically the same delay because of the limited length of the OFDM-like pilot symbol. This requirement results from the discussion presented in Section 3, see also Table 1. Nevertheless, case A is included in our example for possible use with other pilot configurations.

We are making the following assumptions:

- the carrier frequency equals 70 GHz, as before;
- BW of the signal equals 100 MHz;
- the signal is critically sampled, i.e., $f_{s}=$ 100 MHz.

We start by defining the maximum Doppler value based on (21): $f_{d}=14$ kHz. Now, the maximum value of *M* could be defined based on (22). After rounding the obtained value down to the power of 2, the maximum *M* equal to 2048 is obtained.

For $f_{s}=100$ MHz, the size of the resolution element in the range direction is 3 m. In the calculation of $\Delta t_{element}$, the maximum velocity of the use case considered should be used.

In use case B $\Delta t_{element}=50$ ms. To define the number of samples in one RP-OTFS frame, we assume that the time duration of the OTFS frame in (23) should be at least ten times smaller than $\Delta t_{element}$. Thus, the number of samples in one OTFS frame in use case B is equal to 500,000. Rounding this value down to the nearest power of two, one obtains MN = 262,144. The final step is to choose concrete values of *M* and *N*. Based on considerations about computational complexity, discussed before, and on the maximum value *M* calculated in the example, we obtain M=2048, N=128.

The values of the OTFS parameters, together with the resolution values, calculated for the three considered use cases A, B and C, are presented in Table 2.

## 7. Simulation Results

### 7.1. Sinc Extrapolation-Based Data-to-Data Interference Reduction Only

At the very beginning of the simulation section, we present our sinc extrapolation-based interference reduction results, based on Equation (20). For the test, we used the regular RP-OTFS, i.e., without repetition of pilot carriers, but half of the data carriers of the DD grid are inactive, i.e., filled with zeros. We simulate the influence of the transmission channel with fractional delays with respect to the DD grid, as described in Section 4. After that, we estimated the channel and compensated the CIR oscillations by sinc extrapolating. The part of the grid with zeros was used to estimate the magnitude of the remaining interference.

**Figure 13:**
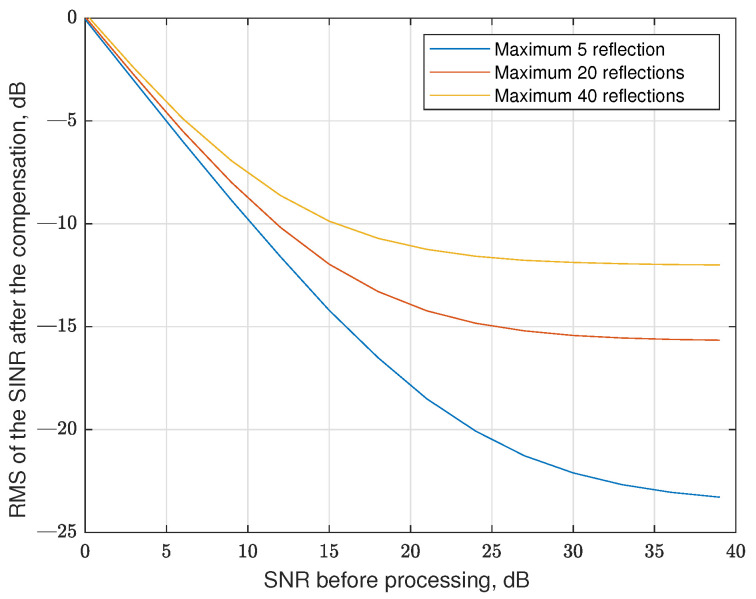
The efficiency of the data-to-data interference compensation in regular RP-OTFS without pilot repetition for different numbers of maximum reflections.

We estimated the dependence of root-mean-square (RMS) of the signal to interference and noise ratio (SINR) on the SNR of the signal before applying the channel, for different numbers of reflections. The reflection coefficient had a normal distribution with the same variance as active data cells, delays had a uniform distribution in the range of 0…L−2, and the Doppler was also distributed uniformly in the range of −0.3·N…0.3·N. The other parameters of the grid had the following values: M=32,N=128,L=8. The results of the simulation are shown in Figure 13.

It is clearly seen in Figure 13 that for high SNR, the sinc extrapolation-based interference reduction algorithm is saturated, which results from the error of estimation of the parameter of the sinc function. Also, in the algorithm we use, we assume that the fractional part of the delay equals 0.5 (coefficient 0.5 in (20)), which is also a source of inaccuracy. We do not know the real delay, so we choose the value in the middle of the possible fractional delay interval.

In the following part of this section, we will estimate by simulation the BER for different application scenarios of regular RP-OTFS without pilot repetition and ZP-OTFS. The OTFS parameters (common for RP and ZP) are given in Table 3. *M* and *N* were reduced to speed up the simulation.

The OFDM waveform simulation was added to the simulation as a baseline. The length of the OFDM symbol was M−L, the length of the CP was *L*. *L* pilots were evenly distributed inside the OFDM symbol. In this case, it was 16 data carriers and 8 pilots per one OFDM symbol.

**Figure 14:**
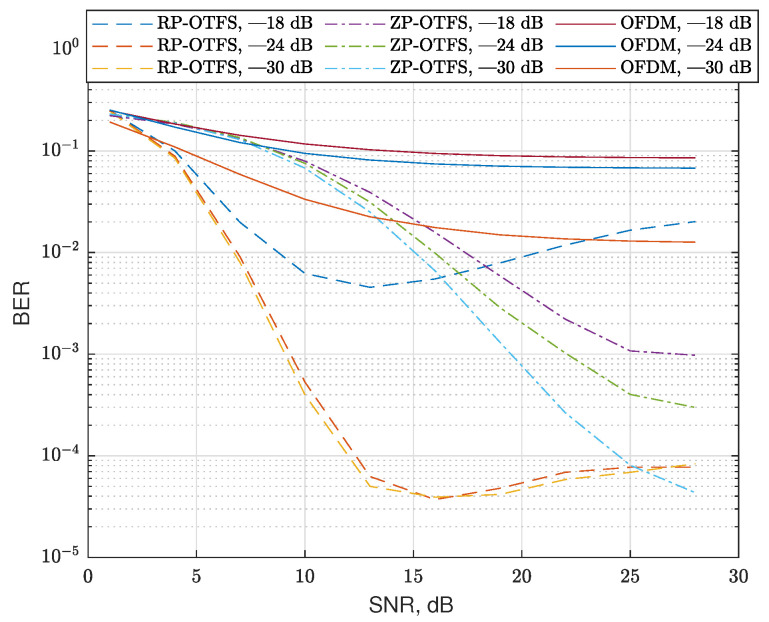
BER for channels with 10 reflections for different modulation types and channel coefficient variances **without** sinc extrapolation-based data-to-data interference compensation. The type of modulation and variance of the channel coefficients are indicated in the legend of the figure.

**Figure 15:**
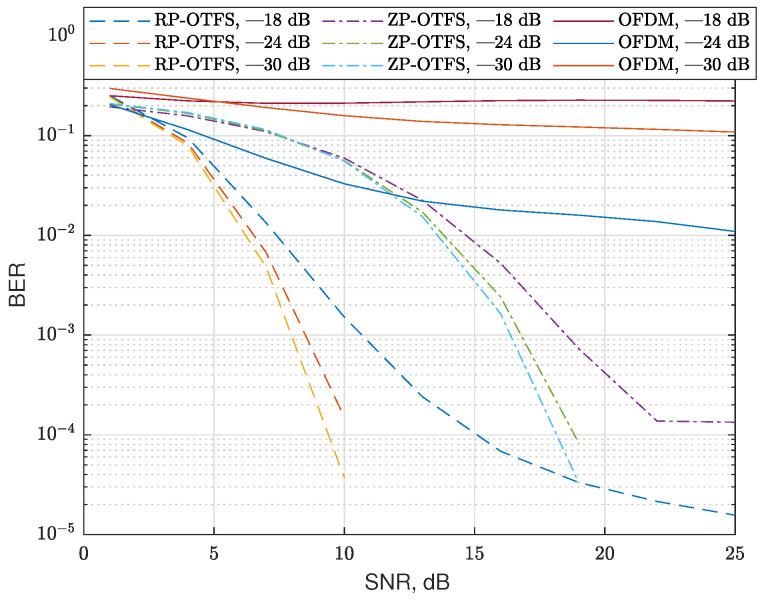
BER for channels with 10 reflections for different modulation types and channel coefficient variances **with** sinc extrapolation-based data-to-data interference compensation. The type of modulation and variance of the channel coefficients are indicated in the legend of the figure.

The first scenario assumes that there is only one path with zero Doppler and 10 randomly scattered fractional reflections. The zero Doppler path is assumed to be the main propagation path, and the receiver is perfectly synchronized to it (no time delay in this path). In this scenario, we fix the number of reflections, but we change the variance of the magnitudes of the CIR coefficients. The variances are normalized to the power of the main propagation path (zero Doppler path). As in the previous case, the CIR coefficients have a normal distribution and zero mean. The delays and Doppler are uniformly distributed in the same range as in the previous case. The results of the simulation without and with the data carrier interference reduction algorithm are presented in Figure 14 and Figure 15, respectively. In this scenario, we used only sinc extrapolating-based data-to-data interference compensation; no pilot repetition is applied.

In the figures, one can see that OFDM is inefficient in such a strong Doppler environment. Also, regular RP-OTFS outperforms ZP-OTFS in case of low SNRs, but for higher SNRs, interference compensation is needed for regular RP-OTFS to outperform ZP-OTFS. In case of lower SNRs, the dominating source of errors is noise, and since RP-OTFS has more active carriers, it is more efficient despite the bigger interference from the pilot zone. As the SNR increases, the interference becomes the dominating factor that determines the efficiency of the system. The interference compensation is not as efficient in the case of ZP-OTFS as it has less power in the pilot zone. One of the obvious solutions is to increase the power of the ZP-OTFS pilot to the level of the regular RP-OTFS pilot zone. However, this will lead to an extremely high peak-to-average ratio, which, in turn, will make the transmission in real systems extremely difficult.

### 7.2. Additional Pilot Repetition-Based Pilot-to-Data Interference Reduction

In the next scenario, sinc extrapolation-based data-to-data interference compensation is always enabled and we compare only the system performance with and without pilot-to-data interference compensation (by the introduced pilot repetition in the Doppler direction of the DD grid), proposed in Section 5 and graphically explained in Figure 12. At present, we simulate a strong clutter reflection. We use Doppler reflection parameters the same as in the previous scenario, but pick only one magnitude −18 dB. However, we also add a strong reflection on zero Doppler, the magnitude of the reflection being −3 dB. The magnitudes of Doppler reflections are normalized to the main line of sight (LOS) propagation path. The maximum delay of the clutter reflections does not exceed L−2. Here, it is important to notice that for moving the transmitter or receiver, the reflections from static objects will also have Doppler. For this case, the parameters of the pilot repetitions *K* and *i* should be chosen to exclude the influence of these strong reflections in the DD domain. In the cases where the pilot repetition was enabled, the column of the DD grid where the interferences were concentrated was excluded from processing, for both types of signal, ZP-OTFS and RP-OTFS. This simulates the case of interference between MTs described in Section 5.

**Figure 16:**
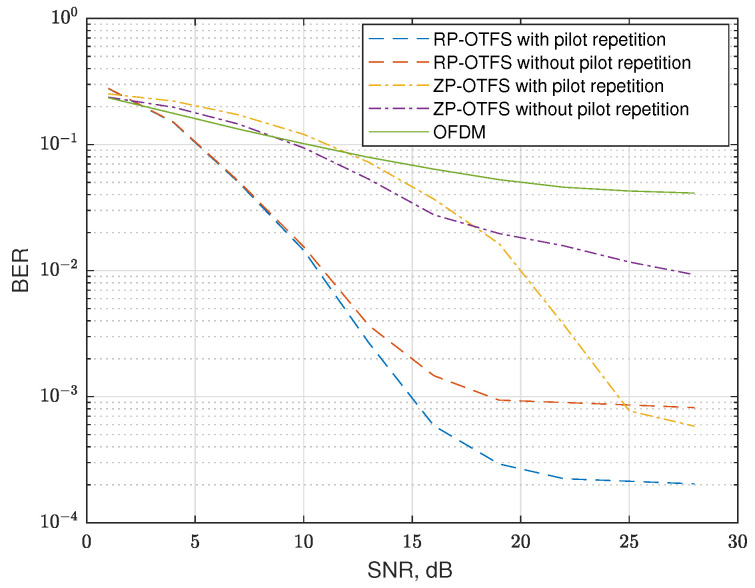
BER for channels with 10 reflections and strong reflection on zero Doppler with enabled and disabled the proposed pilot-to-data interference compensation technique.

The results of the simulation are shown in Figure 16 and are denoted as “with pilot repetition” and “without pilot repetition”, respectively. In the case of strong reflections, the side lobes significantly reduce the performance, even in cases when sinc extrapolation is enabled. The additional pilot-to-data interference compensation technique allows efficient elimination of pilot side-lobes in DD grid regions used by data and reduction in the BER.

### 7.3. Sensing Performance Simulation

**Figure 17:**
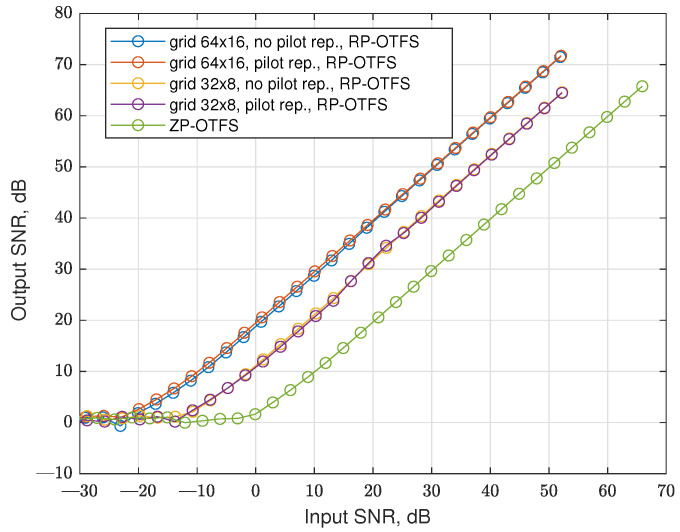
$SNR_{out}$ as a function of $SNR_{in}$ for the factional channel and different modulation techniques.

For radar simulation, we added Doppler to a strong reflection and used a CSI-based approach only, as discussed in [5]. The results obtained are presented in Figure 17. As the data transmission does not influence the radar subsystem and in the CSI-based approach only pilots are used for target detection, we define input SNR as the ratio of the average power of active pilot carriers to noise on the input of the receiver. The output SNR is the average power of the detected peak compared with the average power in the DD grid cells without any targets. As expected, the ZP-OTFS has no processing gain, and the processing gain of the RP-OTFS depends on the number of active pilot carriers.

**Figure 18:**
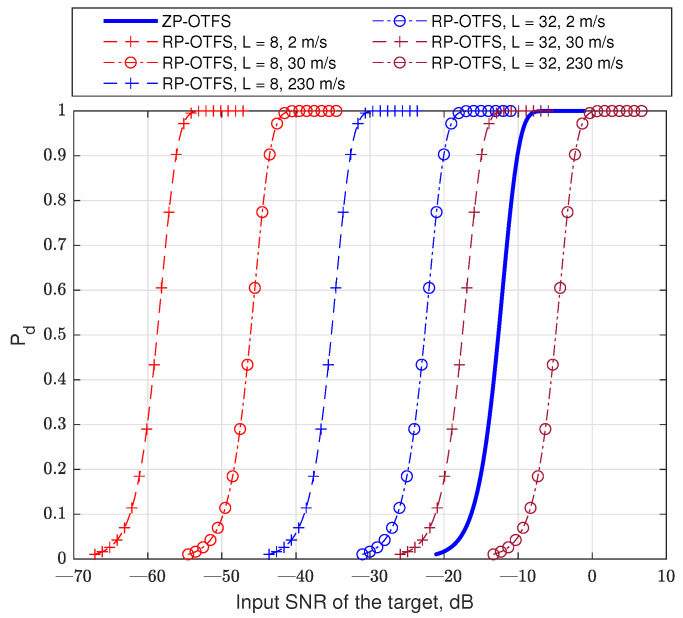
The detection probability in case of constant false alarm. N=128.

Figure 18 presents the simulated probability of the right detection depending on the SNR, for a constant false alarm ration $10^{-4}$ and for different pilot lengths *L* and different velocities of the target. In our simulation, we took into account the interferences between carriers in the OFDM-based pilot. The ZP-OTFS was used as the baseline for our simulations. The performance in the case of ZP-OTFS does not depend on the velocity of the target (until it remains in one resolution cell during the whole OTFS frame). In this simulation, we do not take into account the cell migration of the target.

## 8. Conclusions

In this paper, an important enhancement of RP-OTFS modulation was introduced based on the repetition of random pilot samples. This small modification was shown to have a significant impact on reducing the ICI between pilot carriers (stronger) and data carriers (weaker), and consequently, cause BER reduction (Figure 16) in the ISAC system. An additional step (sinc extrapolation) has been proposed to be added to the OTFS signal processing chain that allows to reduce the level of interference in scenario where a large number of relatively weak reflections are present (Figure 15). The comparison of OFDM, ZP-OTFS, and RP-OTFS without and with these two techniques has demonstrated that the methods proposed here offer the smallest BER for a carrier frequency of 70 GHz. The ability of the dynamic to change the location of the pilot interference in the Doppler direction allows adjusting the grid to different Dopplers without changing processing on the receiver side.

At the same time, as was shown, the new method also allows for a more noise-robust detection of moving vehicles in mm-wave communication (Figure 17). Finally, it was shown how to choose the parameters of the RP-OTFS for efficient work in the millimeter wave bandwidth (Table 2). 

## Figures and Tables

**Table 1:** Maximum operating velocity of the 70 GHz RP-OTFS.

	−40 dB Threshold		−50 dB Threshold	
*L*	$f_{d}$, Hz	v, km/h	$f_{d}$, Hz	v, km/h
8	20,000	431	6900	148
16	9930	214	3089	67
32	5000	108	1567	34
64	2465	53	0	0
128	1336	29	0	0

**Table 2:** The parameter of the RP-OTFS-based radar (70 GHz, BW 100 MHz, $L=\frac{M}{4}$).

Speed	*N*	*M*	Velocity [m/s]		Range [m]	
			**Resolution**	**Max/Min**	**Resolution**	**Max/Min**
A	128	512	2.65	±170	3	384
B	128	2048	0.47	±30	3	1534
C	128	65,536	0.01	±0.64	3	49,152

**Table 3:** Values of simulation parameters.

Parameter	Value	Description
Modulation	4-QAM	Type of carrier modulation
$f_{c}$	70 GHz	Carrier frequency
$f_{s}$	100 MSPS	Sampling rate
*M*	32	No of samples in delay direction
*N*	16	No of samples in Doppler direction
*L*	8	Number of pilot samples

## Data Availability

The original contributions presented in the study are included in the article, further inquiries can be directed to the corresponding author.
